# Clinical outcomes of complete cytoreduction with concurrent liver resection followed by hyperthermic intraperitoneal chemotherapy for synchronous peritoneal and liver metastatic colorectal cancer

**DOI:** 10.1186/s12957-019-1746-x

**Published:** 2019-12-11

**Authors:** Youngbae Jeon, Eun Jung Park, Jin Hong Lim, Seung Hyuk Baik

**Affiliations:** 1 Division of Colon and Rectal Surgery, Department of Surgery, Gangnam Severance Hospital, Yonsei University College of Medicine, 20, Eonju-ro 63-gil, Gangnam-gu, Seoul, 06229 Korea; 2 Division of Hepatobiliary and Pancreatic Surgery, Department of Surgery, Gangnam Severance Hospital, Yonsei University College of Medicine, Seoul, Korea

**Keywords:** Colorectal cancer, Peritoneal metastases, Liver metastases, Cytoreductive surgery, Hyperthermic intraperitoneal chemotherapy, Liver resection

## Abstract

**Background:**

This study aimed to evaluate the clinical outcomes of concurrent liver resection with cytoreductive surgery and hyperthermic intraperitoneal chemotherapy in colorectal cancer patients with synchronous liver and peritoneal metastases.

**Methods:**

Patients with colorectal liver and peritoneal metastasis who underwent complete cytoreduction and hyperthermic intraperitoneal chemotherapy with concurrent liver surgery between September 2014 and July 2018 were included. Perioperative outcomes, overall survival, and progression-free survival were analyzed retrospectively.

**Results:**

In total, 22 patients were included. The median peritoneal cancer index was 13 (range, 0–26), and the median number of liver metastases was 3 (range, 1–13). The mean total operative time was 11.4 ± 2.6 h. Minor postoperative complications (Clavien-Dindo grade I–II) were reported in 10 patients (45.5%), and major postoperative complications (grade III–V) were reported in five patients (22.7%), including one mortality patient. The median overall survival since diagnosis with metastasis was 27.4 months. The median overall survival since surgical intervention and the progression-free survival were 16.7 months and 7.1 months, respectively.

**Conclusions:**

This short-term follow-up study showed that, in an experienced center, combined resection with hyperthermic intraperitoneal chemotherapy for colorectal liver and peritoneal metastases was feasible and safe with acceptable oncologic outcomes.

## Background

Approximately 20–25% of colorectal cancer (CRC) patients are diagnosed with metastatic CR C[1]. The most common site of metastatic CRC is the liver, accounting for 30–40% of metastatic CRC cases, and the most common cause of death is liver metastasis followed by peritoneal metastasis [2, 3]. Complete resection with systemic chemotherapy is a potentially curative treatment option for patients with metastatic CRC. Complete liver resection of hepatic metastasis resulted in a median overall survival (OS) of 30–50 months, and 5-year survival of up to 50% [4]. The development of surgical techniques, such as two-stage hepatectomy and portal vein embolization, can increase the resectability rate even in patients with multiple, initially unresectable, liver metastases [5, 6]. For peritoneal metastasis, hyperthermic intraperitoneal chemotherapy (HIPEC) after cytoreductive surgery (CRS) is effective for patients who can achieve complete cytoreduction. Previous studies, including a randomized trial, showed better survival outcomes and acceptable morbidity rates in highly selected patients who were treated with CRS and HIPEC [2, 7–10]. Although recently PRODIGE-7 trial suggested that the addition of HIPEC with oxaliplatin does not influence the survival results, it did not evaluate HIPEC using mitomycin, and the survival benefit with HIPEC was found in a subgroup with medium extent of peritoneal carcinomatosis.

However, the inclusion criterion for resection of metastatic CRC is usually that the metastatic lesion is confined to a single organ or structure. Combined metastatic CRC patients are generally recommended palliative systemic chemotherapy. Patients with combined metastatic sites, especially those including peritoneal metastasis, have shorter OS [11]. When systemic chemotherapy was the only administered treatment, the median OS of patients with peritoneal metastasis combined with other sites of metastasis was 12.6 months, whereas the median OS of patients with solitary peritoneal metastasis was 16.3 months [12]. The incidence of synchronous peritoneal and liver metastases is estimated to be 8% of metastatic CRC cases, with OS of only 2.6 months when patients do not receive chemotherapy and OS of 12.0 months when patients receive chemotherapy [13]. Therefore, due to this poor prognosis, several physicians have attempted surgical treatment for patients with combined peritoneal and hepatic metastases, despite the development of chemotherapeutic agents. In this study, we aimed to evaluate the clinical outcomes of CRS and HIPEC with concurrent liver surgery for patients with synchronous liver and peritoneal metastatic CRC.

## Methods

### Patient selection

From September 2014 to July 2018, patients who underwent CRS and HIPEC with concurrent liver surgery at Gangnam Severance Hospital, Yonsei University College of Medicine, Seoul, Republic of Korea were retrospectively reviewed. We included patients diagnosed with synchronous liver and peritoneal metastases, Eastern Cooperative Oncologic Group performance status score of < 2, and age < 80 years. We excluded patients who diagnosed with non-colorectal origin cancer, other extrahepatic hematogenous metastasis, and incomplete cytoreduction, indicated by a completeness of cytoreduction (CC) score of 2 or 3. Concurrent liver surgery included any type of liver resection, two-stage hepatectomy, and intraoperative radiofrequency ablation. All patients were preoperatively diagnosed with metastatic CRC by abdomino-pelvic computed tomography (CT), liver magnetic resonance imaging, or positron emission tomography. A multidisciplinary team discussed the appropriate treatment plan and eligibility for surgery. All data of consecutive patients were retrospectively collected and reviewed using electronic medical records system. This study was approved by the Institutional Review Board of Gangnam Severance Hospital, Yonsei University College of Medicine (IRB No. 3-2019-0005).

### Assessment parameters

Baseline demographics, including age, sex, body mass index (BMI), the American Society of Anesthesiologists (ASA) classification, history of previous abdominal surgery, history of preoperative chemotherapy, preoperative laboratory findings, biomarker, and characteristics of primary cancer, were collected. The PCI and CC scores were assessed intraoperatively, according to the techniques given by Sugarbaker et al [14]. The number and the largest diameter of the liver metastases were measured via liver magnetic resonance imaging. Postoperative complications within 30 days were evaluated by the Clavien-Dindo classification [15]. Readmission was defined as admission after discharge due to surgery-related complications. Postoperative laboratory findings, except tumor markers, were collected on the operative day, and postoperative tumor markers were checked 1 week after the last operation.

OS was calculated from the date of surgical intervention (in case of two-stage hepatectomy, from the date of first surgical intervention) until the date of death or last follow-up. However, in order to include the impact of preoperative chemotherapy and compensate for the long period from diagnosis to surgery, OS was recalculated from the date of diagnosis of metastatic CRC. Progression-free survival (PFS) was measured from the date of surgical intervention (in case of two-stage hepatectomy, from the date of first surgical intervention) until the date of first recurrence or last follow-up.

### Surgical technique

Intraperitoneal exploration was initially performed via midline laparotomy. After assessing the extent of intraoperative peritoneal cancer, cytoreduction was performed according to the Sugarbaker technique [16]. Selective peritonectomy was performed depending on the site of peritoneal metastases, and resection of the bowel or intraperitoneal organs was performed when the seeding tumor showed gross invasion. After cytoreduction, concurrent liver surgery, including liver resection and/or intraoperative radiofrequency ablation, was performed. Intraoperative radiofrequency ablation was performed to treat complex liver metastases which are unsuitable for parenchymal resection alone. Some of the selected patients underwent two-stage hepatectomy when they had multiple liver metastases that could not be completely resected via single-stage hepatectomy, considering the remnant liver volume. The eligibility of two-stage hepatectomy was determined by a multidisciplinary team. Two-stage hepatectomy was complete when patients showed resectable disease after the first-stage liver surgery, which included portal vein ligation. HIPEC was performed using mitomycin C (35 mg/m^2^ at 42–43 °C) mixed in 3 L of hypertonic solution (Dianeal®, 1.5% dextrose peritoneal dialysis solution) using the open Coliseum technique for 90 min. Mitomycin C (17.5 mg/m^2^) was initially administered, followed by additional administration of 8.8 mg/m^2^ at 30 and 60 min, respectively. The resected bowel was reconstructed after HIPEC.

### Follow-up

Patients visited the outpatient clinic 1 week after being discharged. Postoperative chemotherapy was initiated 1 week after the first visit, if a patient had no systemic complications. Patients were followed up by using serum tumor marker measurements, abdomino-pelvic CT, and chest CT at 3-month intervals. If tumor recurrence was suspected on regular follow-up imaging, positron emission tomography or liver magnetic resonance imaging was selectively performed.

### Statistical analysis

Perioperative outcomes, OS, and PFS were analyzed using SPSS 23 (SPSS Inc., Chicago, IL). Survival time was estimated using the Kaplan-Meier method, and reported along with the confidence interval (CI).

## Results

### Baseline patient characteristics

A total of twenty-two patients (10 men, 12 women; median age, 56 years [range, 26–66 years]) were included in this study. The median BMI was 23.2 kg/m^2^, and five patients (22.7%) had an ASA score of 3. Fourteen patients (63.6%) had a history of previous abdominal surgery, and 12 patients (54.5%) preoperatively underwent primary tumor resection. Sigmoid colon was the most common site of primary CRC (*n* = 8, 36.4%), and moderate differentiation was the most common histologic grade (*n* = 20, 90.9%). Twenty patients (90.9%) received preoperative chemotherapy. The median value of preoperative CEA and CA 19-9 were 11.9 ng/mL and 34.7 U/mL, respectively. The median preoperative aspartate aminotransferase (AST) and alanine aminotransferase (ALT) were 25.5 IU/L and 21.0 IU/L, respectively. Fourteen patients (63.6%) had K-ras mutation. The median number of liver metastases was 3 (range, 1–13), and the median value of the largest diameter of liver metastases was 1.4 cm (range, 1–5 cm) Fifteen patients had liver metastases in the right lobe, two patients had in the left lobe, and five patients had in both lobes (Table 1).

**Table 1:** Baseline patient characteristics

Variables	Total patients (*n*=22)
Age^‡^ , years	56 (26-66)
Sex*	
Male : Female	10 (45.5) : 12 (54.5)
BMI^‡^, kg/m^2^	23.2 (16.2-32.2)
ASA score*	
1	5 (22.7)
2	12 (54.5)
3	5 (22.7)
History of previous abdominal surgery*	14 (63.6)
Preoperative resection of primary cancer*	12 (54.5)
Primary cancer origin*	
Cecal cancer	2 (9.1)
Ascending colon cancer	5 (22.7)
Descending colon cancer	2 (9.1)
Sigmoid colon cancer	8 (36.4)
Rectosigmoid junction cancer	1 (4.5)
Rectal cancer	4 (18.2)
Primary cancer histologic grade*	
well-differentiated	0
moderate-differentiated	20 (90.9)
poorly-differentiated	1 (4.5)
Mucinous	0
signet ring cell	0
Unknown	1 (4.5)
Preoperative chemotherapy*	20 (90.9)
1st line	16 (72.7)
2nd line	3 (13.6)
3rd line	1 (4.5)
Preoperative laboratory findings^‡^	
CEA , ng/mL	11.9 (0.6-408.6)
CA 19-9 , U/mL	34.7 (0.8-2606.8)
Hemoglobin , g/dL	12.7 (9.8-14.5)
Albumin , g/dL	4.2 (3.5-4.5)
Total bilirubin , mg/dL	0.6 (0.3-1.2)
AST , IU/L	25.5 (17-85)
ALT , IU/L	21 (9-56)
Biomarker*	
K-ras mutation	14 (63.6)
K-ras wild type	8 (36.4)
Number of liver metastases^‡^	3 (1-13)
The largest diameter of liver metastases^‡^, cm	1.4 (1-5)
Location of liver metastases*	
Right lobe	15 (68.2)
Left lobe	2 (9.1)
Both lobes	5 (22.7)

*, *n* (%); ^‡^, median (range); *BMI* body mass index, *ASA* American Society of Anesthesiologists, *AST* aspartate aminotransferase, ALT alanine aminotransferase

### Detailed surgical procedures

**Fig. 1:**
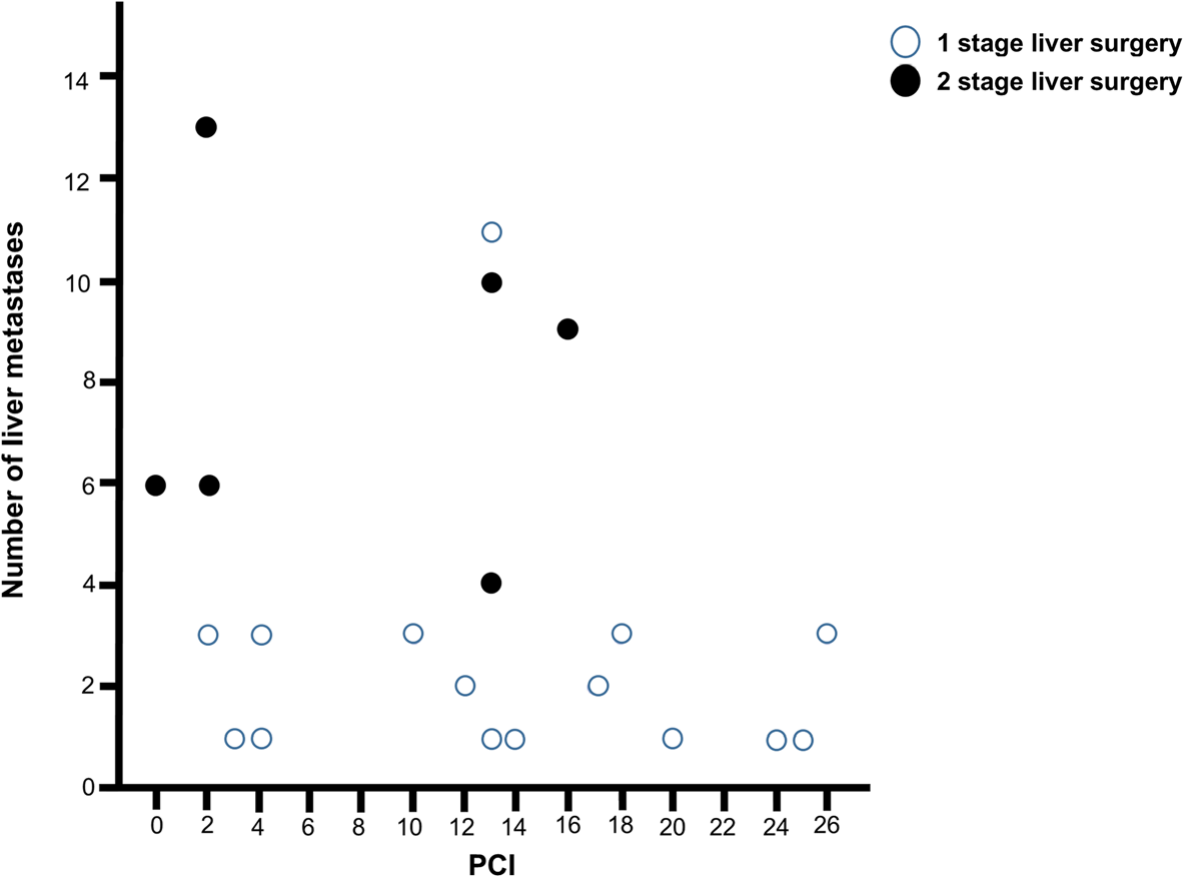
Scatter plot showing the relation between peritoneal cancer index and number of liver metastasis. PCI peritoneal cancer index

Detailed surgical procedures for cytoreductive surgery with concurrent liver surgery was demonstrated in Table 2. Various cytoreductive procedures, such as peritonectomy, bowel resection, hysterectomy, splenectomy, and cholecystectomy, were performed. Omentectomy was the most frequently performed cytoreductive procedure (*n* = 15, 68.2%), and one patient underwent excision of the ureter with ureterocystostomy due to tumor invasion of the right ureter. Wedge resection (*n* = 14, 63.6%) was the most common procedure for concurrent liver surgery. Four patients (18.2%) underwent intraoperative radiofrequency ablation, and two patients underwent intraoperative radiofrequency alone as a replacement for surgical resection. Six patients (27.3%) underwent two-stage hepatectomy. Among them, three patients underwent first-stage liver surgery, followed by CRS and HIPEC with concurrent complete hepatectomy; the other three patients underwent CRS and HIPEC with concurrent liver surgery, followed by complete hepatectomy. All of the patients with > 3 liver metastases underwent two-stage hepatectomy, except for one patient (Fig. 1); although that patient had 11 liver metastases, they were completely resected via multiple wedge resections during single-stage hepatectomy. The mean duration between first-stage liver surgery and second-stage liver surgery was 23.3 ± 17.3 days (range, 12–58 days).

**Table 2:** Detailed surgical procedures for cytoreductive surgeries with concurrent liver surgeries

Variables	Total patients (*n*=22)
Detailed procedures for cytoreductive surgery	
Peritoneum	
Diaphragmatic peritonectomy	11 (50.0)
Parietal peritonectomy	8 (36.4)
Pelvic peritonectomy	13 (59.1)
Omentectomy	15 (68.2)
Gastro-intestinal tract	
Right hemicolectomy	3 (13.6)
Left hemicolectomy	2 (9.1)
Anterior resection	3 (13.6)
Low anterior resection	8 (36.4)
Ileocecectomy	2 (9.1)
Segmental resection of small bowel	8 (36.4)
Hartmann operation	1 (4.5)
Appendectomy	3 (13.6)
Gynecologic	
Hysterectomy	5 (22.7)
Salpingo-oophorectomy	8 (36.4)
Others	
Excision of ureter with ureterocystostomy	1 (4.5)
Cholecystectomy	8 (36.4)
Splenectomy	1 (4.5)
Detailed procedures of liver surgery	
Intraoperative radiofrequency ablation	4 (18.2)
Wedge resection	14 (63.6)
Sectionectomy	1 (4.5)
Lobectomy	4 (18.2)
Extended lobectomy	1 (4.5)
Two-stage hepatectomy	6 (27.3)
1^st^-stage liver surgery → CRS/HIPEC + 2^nd^-stage liver surgery	3 (13.6)
CRS/HIPEC + 1^st^-stage liver surgery → 2^nd^-stage liver surgery	3 (13.6)

*n* (%); *CRS* cytoreductive surgery, *HIPEC* hyperthermic intraperitoneal chemotherapy

### Perioperative outcomes

**Fig. 2:**
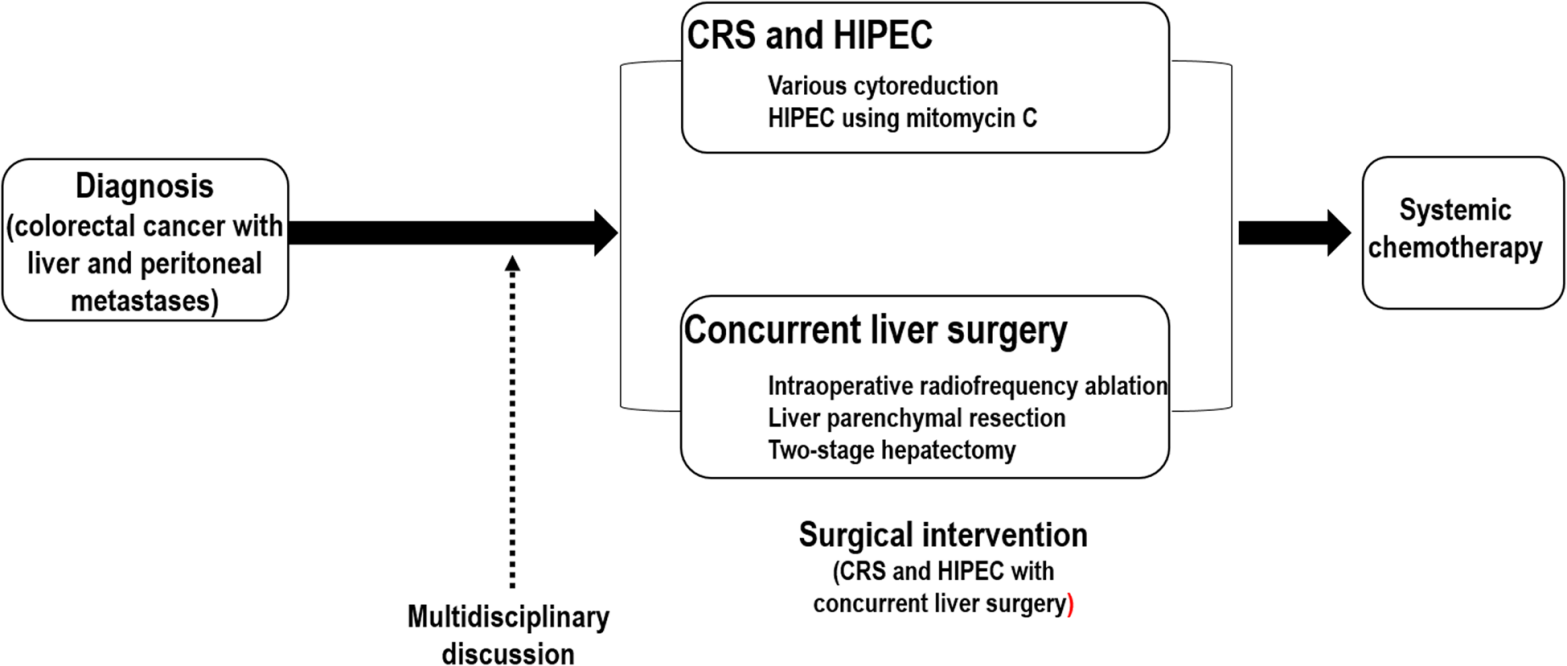
Schematic flow diagram showing treatment. CRS cytoreductive surgery, HIPEC hyperthermic intraperitoneal chemotherapy

The median PCI was 13 (range, 0–26), and the distribution of PCI was as follows: PCI < 10, *n* = 7 (31.8%); PCI 10–19, *n* = 11 (50.0%); and PCI ≥ 20, *n* = 4 (18.2%). All of cytoreductive surgery was achieved CC-0. The mean total operative time was 11.4 ± 2.6 h (range, 7.5–16.5 h), and the mean operative time for concurrent liver surgery was 3.0 ± 2.5 h (range, 0.5–9.0 h). The mean estimated blood loss was 1418.6 ± 1204.1 mL (range, 250–5000 mL) and the mean intraoperative transfusion was 475.4 ± 740.4 mL (range, 0–3030 mL). The median postoperative CEA and CA 19-9 were 2.6 ng/mL and 29.4 U/mL. The median postoperative AST and ALT were 170.5 IU/L and 137 IU/L. The mean lengths of intensive care unit stay and hospital stay were 3.1 ± 7.9 days and 25.6 ± 16.7 days, respectively. All of the patients, except for one patient who died, received postoperative chemotherapy; and the median interval between surgery and adjuvant chemotherapy was 40.5 days (range, 31–63). Table 3 shows perioperative outcomes, and Fig. 2 shows a schematic flow diagram of the treatment.

**Table 3:** Perioperative outcomes

Variables	Total patients (n=22)
Peritoneal cancer index (PCI)*	
<10	7 (31.8)
10-19	11 (50.0)
≥20	4 (18.2)
Mean PCI^†^	12.0 ± 7.9 (0-26)
Median PCI ^‡‡^	13 (3-17)
Completeness of cytoreduction (CC)	
CC-0	22 (100)
CC-1	0
Total operative time^†^, hour	11.4 ± 2.6 (7.5-16.5)
Operative time for concurrent liver surgery^†^, hour	3.0 ± 2.5 (0.5-9.0)
Estimated blood loss^†^, mL	1418.6 ± 1204.1 (250-5000)
Intraoperative transfusion^†^, mL	475.4 ± 740.4 (0-3030)
Postoperative laboratory findings^‡^	
CEA , ng/mL	2.6 (0.4-32.9)
CA 19-9 , U/mL	29.4 (4.1-232)
Hemoglobin , g/dL	10.6 (7.5-13.7)
Albumin , g/dL	2.7 (1.4-3.3)
Total bilirubin , mg/dL	1.3 (0.4-2.4)
AST , IU/L	170.5 (77-498)
ALT , IU/L	137 (30-440)
Length of ICU stay^‡^, days	3.1 ± 7.9 (0-38)
Length of hospital stay^‡^, days	25.6 ± 16.7 (9-71)
Numbers to treat adjuvant chemotherapy*	21 (95.5)
Interval between surgery and adjuvant chemotherapy^‡‡^, days	40.5 (31-63)

*, n (%); ^†^, mean ± standard deviation (range); ^‡^, median (range); ^‡‡^, median (quartile); *AST* aspartate aminotransferase; *ALT* alanine aminotransferase; *ICU* intensive care unit

### Postoperative complications

Minor postoperative complications (grade I–II: including neutropenia, anemia, fever, diarrhea, and vomiting) were observed in 10 patients (45.5%). Major postoperative complications (grade III–V: including luminal bleeding, bile leakage, anastomotic leakage, and septic shock), were observed in five patients (22.7%). Three patients (13.6%) underwent reoperation due to surgical complications of bile leakage in one patient and anastomotic leakage in two patients. Of the three patients who underwent reoperation, one patient (4.5%) died 32 days after surgery, despite reoperation for panperitonitis due to anastomotic leakage. The rate of readmission due to postoperative complications was 9.1%, with intestinal obstruction and anastomotic leakage in one patient each (Table 4).

**Table 4:** Postoperative complications

Variables	Total patients (n=22)
Postoperative complications*	
Grade I	1 (4.5)
Grade II	9 (40.9)
Grade IIIa	2 (9.1)
bile leakage	1 (4.5)
luminal bleeding	1 (4.5)
Grade IIIb	1 (4.5)
anastomosis leakage	1 (4.5)
Grade IV	1 (4.5)
septic shock	1 (4.5)
Grade V	1 (4.5)
Reoperation, during postoperative hospital stay	3 (13.6)
Bile leakage	1 (4.5)
Anastomosis leakage	2 (9.1)
Readmission due to postoperative complication	2 (9.1)
Intestinal obstruction	1 (4.5)
Anastomosis leakage	1 (4.5)

*n* (%); * classified by the Clavien-Dindo classification

### Survival outcomes

**Fig. 3:**
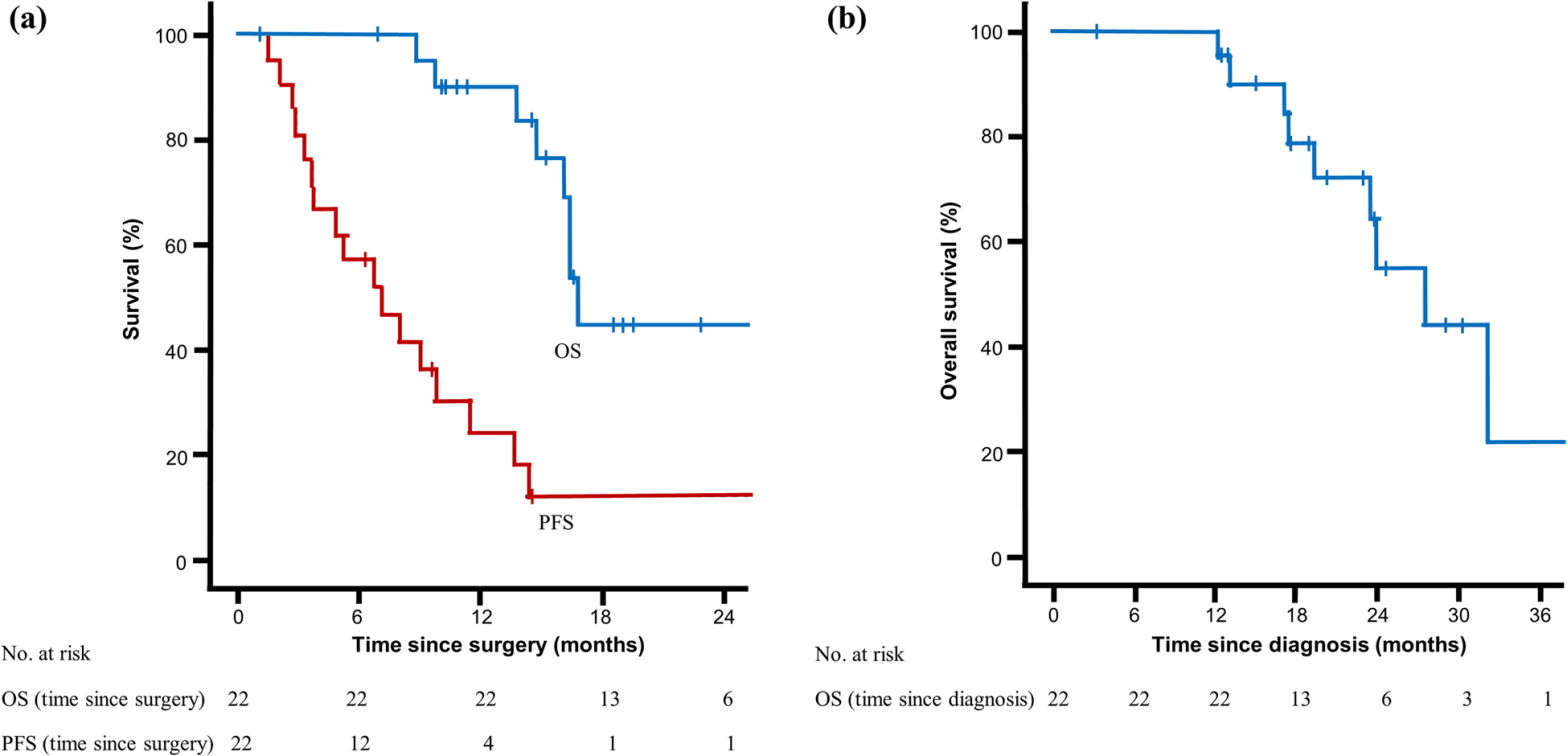
Overall survival and progression-free survival curves (Kaplan-Meier method). **a** Survival rate since surgery. **b** Overall survival since diagnosis of metastatic colorectal cancer. OS overall survival, PFS progression-free survival

**Fig. 4:**
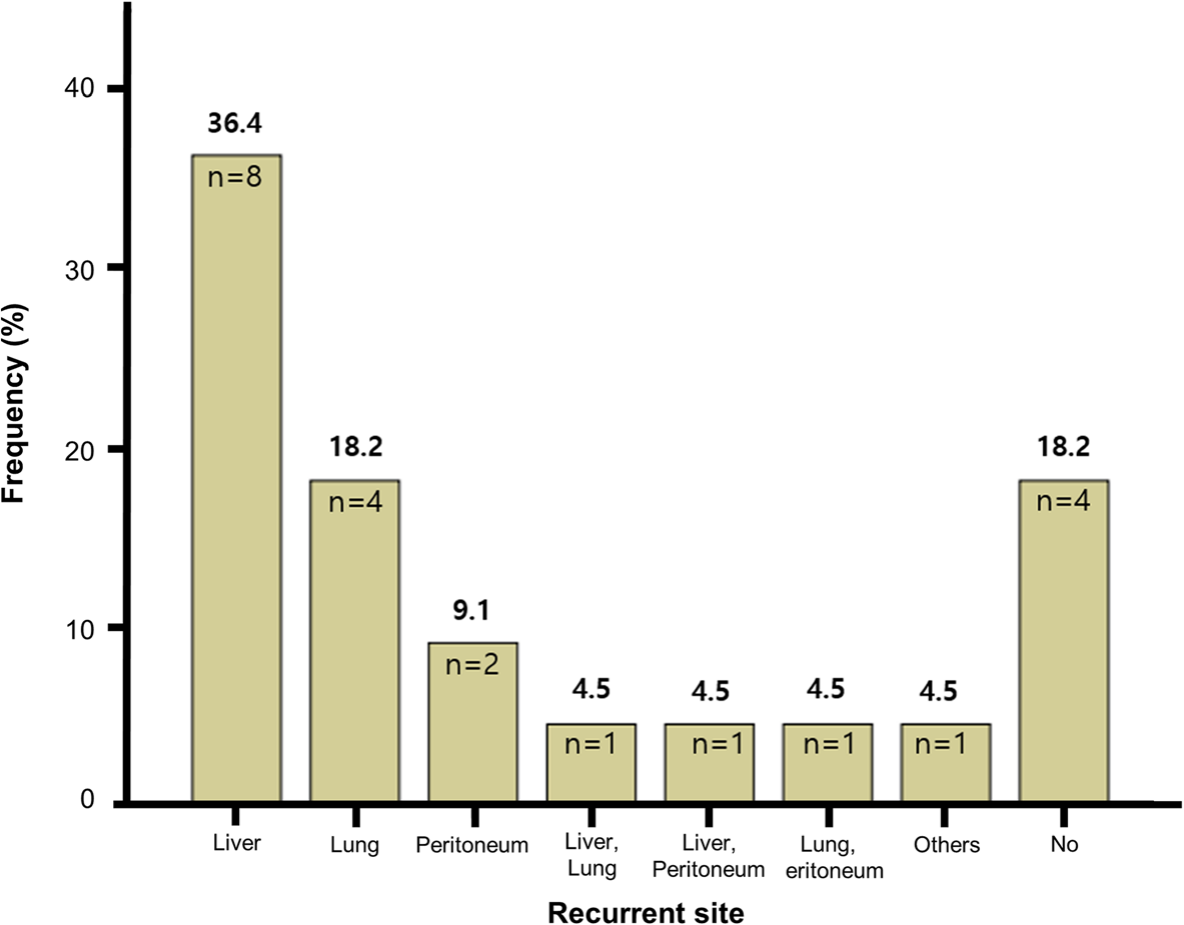
Sites of recurrence after cytoreductive surgery and hyperthermic intraperitoneal chemotherapy with concurrent liver surgery

The median follow-up period was 15.0 months. The median OS and median PFS since surgical intervention were 16.7 months (95% CI, 16.0–17.4 months) and 7.1 months (95% CI, 3.2–11.0 months), respectively. The median OS since diagnosis was 27.4 months (95% CI, 21.7–33.1 months) during a median follow-up of 19.7 months. The 1-year OS was 100%, 2-year OS was 55.0%, 1-year PFS was 24.2%, and 2-year PFS was 12.1% (Fig. 3). The liver was the most frequent site of initially detected recurrence (*n* = 10, 45.5%), followed by the lungs (*n* = 6, 27.3%) and peritoneum (*n* = 4, 18.2%) (Fig. 4). Four patients did not experience recurrence during the follow-up period.

## Discussion

Surgical treatment for patients who have peritoneal metastasis combined with the presence of parenchymal liver metastasis has not been established. Since Elias et al. published the first report about combined resection with intraperitoneal chemotherapy for patients with peritoneal and liver metastases, several surgeons have tried to attempt this challenging surgical treatment [17]. A recently published systematic review showed that combined curative resection with intraperitoneal chemotherapy for peritoneal and liver metastatic CRC resulted in a possible survival benefit in selected patients [18]. However, there have been only a few studies regarding this treatment, therefore the feasibility and the efficacy is still controversial.

Our primary aim was to evaluate the clinical outcomes of combined curative resection for synchronous peritoneal and liver metastases. With comparison of other published studies, our study showed comparable perioperative outcomes. In our study, minor complication rate was 45.5% (*n* = 10), major complication rate was 22.7% (*n* = 5), and mortality rate was 4.5% (*n* = 1). Bile leakage occurred in only two patients (9.1%) who were treated with endoscopic biliary drainage and reoperation, respectively. These results were comparable to those in previously published studies that had major complication rates of 23.8–51.4% and mortality rates of 0–8.1% [3, 19–24].As CRS with HIPEC has an overall morbidity rate of 23–45% and mortality rate of 0–12% in CRC patients with peritoneal metastasis only, the addition of concurrent liver resection does not seem to reduce safety [25]. Berger et al. suggested that perioperative complication rate, length of hospital stay, and intraoperative blood transfusion were related to more extensive cytoreduction, rather than liver surgery itself [26]. In our study, only one patient died of intestinal anastomotic leakage; she had three anastomoses, which was relatively higher compared to other patients, without stoma diversion. A protective stoma is recommended for extensive cytoreduction including ≥ 2 anastomoses to reduce the anastomotic leak rate [27].

Our study showed that the median OS since surgery was 16.7 months, and that since diagnosis was 27.4 months. Downs-Canner et al. reported a median OS of 13.0 months since surgery in patients who were treated with combined resection for colorectal peritoneal and liver metastases, while the median OS was 32.5 months since diagnosis; these findings were comparable to our results [3]. Lorimier et al. also calculated OS from diagnosis, and reported a median OS of 36.1 months; however, only 63% of patients (*n* = 14) were treated with preoperative chemotherapy [24]. Maggiori et al*.* reported a median OS of 32.0 months, although they measured survival since surgery [19]. They excluded patients with disease progression within 2–3 months after receiving preoperative chemotherapy; we also included such patients. Therefore, in our study, four patients received more than first-line preoperative chemotherapy, and the average interval between diagnosis and surgery was 6.5 months. Maggiori et al. suggested that better survival prognosis could be achieved when patients had PCI < 12, and the number of liver metastases was < 3 [19]. The median OS was 40 months for patients with PCI < 12 and < 3 liver metastases, while it was 27 months for patients with PCI ≥ 12 and ≥ 3 liver metastases [19]. In our study, only two patients met these criteria for better survival prognosis, and both of them were alive for 30.2 months and 20.2 months until the date of the last follow-up. Carvalho et al. showed relatively better survival outcomes, with a median OS of 44 months and median PFS of 10 months. These better results might be due to the fact that the patients had relatively lower PCI (median, 5; range, 3–10.5) and fewer liver metastases (median, 2; range, 1–6) [20]. Our results for OS were acceptable, despite the longer period for preoperative chemotherapy including patients with disease progression and more severe liver metastases.

In our study, the median PFS was 7.1 months, which was comparable to that obtained in previous studies (5.1 to 10.0 months) [3, 19–24]. The most common site of initial recurrence was the liver (*n* = 10, 45.5%). Recently, Ito et al. suggested that hepatectomy for metachronous colorectal liver metastases after complete CRS and HIPEC for peritoneal metastases could be performed safely and achieved better survival outcome [28]. However, in our study, since the patients had synchronous peritoneal and hepatic metastases, it would be challenging to apply repeated hepatectomy for patients who had liver recurrence after CRS and HIPEC with concurrent liver surgery. According to Ito et al., 75% of patients with metastatic CRC, who underwent hepatectomy with curative intent, experienced recurrence in the liver before they died [4]. Moreover, one-third of the patients ultimately showed systemic dissemination with bone or brain metastases. This implies that even if complete resection is successful, systemic chemotherapy is needed for CRC patients with hepatic metastasis.

We included six patients who underwent two-stage hepatectomy, a relatively more aggressive treatment strategy compared to that performed in previous studies. Since Adam et al. reported the first series on two-stage hepatectomy in patients with initially unresectable colorectal liver metastasis, several studies have suggested that two-stage hepatectomy was safe and effective for selected patients [6, 29]. According to these studies, the morbidity and mortality rates of two-stage hepatectomy for multiple colorectal liver metastases ranged from 26 to 59% and from 0 to 9%, respectively [30–34]. Although only six patients in our study underwent two-stage hepatectomy, it was encouraging to see that only one patient experienced bile leakage (grade IIIa), no patient showed liver failure, and no patient died after CRS and HIPEC with concurrent two-stage hepatectomy. However, to analyze the safety and effectiveness of this procedure, much more patients are required and the patient selection should be carefully taken. Further study regarding two-stage hepatectomy will be conducted.

This study had several limitations, including its small patient population, retrospective nature of analysis, and short follow-up period. However, the study results showed acceptable morbidity and mortality rates with comparable survival outcomes, even though patient selection was relatively more extensive compared to previous studies. Therefore, after refining the patient selection criteria through further studies, this strategy could be effective for CRC liver and peritoneal metastases.

## Conclusions

In an experienced center, concurrent liver resection with CRS and HIPEC was feasible and safe for selected patients, with acceptable clinical outcomes. Nevertheless, additional long-term follow-up studies on a large population are needed to confirm the effectiveness of the surgical treatment for patients with liver and peritoneal metastatic CRC.

## Data Availability

All data generated or analyzed in this study are included in the published article.

## Abbreviations

CC: Completeness of cytoreduction CRC Colorectal cancer CRS Cytoreductive surgery HIPEC Hyperthermic intraperitoneal chemotherapy OS Overall survival PCI Peritoneal cancer index PFS Progression-free survival
